# *Trichophyton rubrum* is Inhibited by Free and Nanoparticle Encapsulated Curcumin by Induction of Nitrosative Stress after Photodynamic Activation

**DOI:** 10.1371/journal.pone.0120179

**Published:** 2015-03-24

**Authors:** Ludmila Matos Baltazar, Aimee E. Krausz, Ana Camila Oliveira Souza, Brandon L. Adler, Angelo Landriscina, Tagai Musaev, Joshua D. Nosanchuk, Adam J. Friedman

**Affiliations:** 1 Department of Microbiology & Immunology, Albert Einstein College of Medicine, Bronx, New York, United States of America; 2 Division of Dermatology, Department of Medicine, Albert Einstein College of Medicine, Bronx, New York, United States of America; 3 Department of Physiology and Biophysics, Albert Einstein College of Medicine, Bronx, New York, United States of America; 4 Division of Infectious Diseases, Department of Medicine, Albert Einstein College of Medicine, Bronx, New York, United States of America; Albany Medical College, UNITED STATES

## Abstract

Antimicrobial photodynamic inhibition (aPI) utilizes radical stress generated from the excitation of a photosensitizer (PS) with light to destroy pathogens. Its use against *Trichophyton rubrum*, a dermatophytic fungus with increasing incidence and resistance, has not been well characterized. Our aim was to evaluate the mechanism of action of aPI against *T*. *rubrum* using curcumin as the PS in both free and nanoparticle (curc-np) form. Nanocarriers stabilize curcumin and allow for enhanced solubility and PS delivery. Curcumin aPI, at optimal conditions of 10 μg/mL of PS with 10 J/cm^2^ of blue light (417 ± 5 nm), completely inhibited fungal growth (p<0.0001) via induction of reactive oxygen (ROS) and nitrogen species (RNS), which was associated with fungal death by apoptosis. Interestingly, only scavengers of RNS impeded aPI efficacy, suggesting that curcumin acts potently via a nitrosative pathway. The curc-np induced greater NO^•^ expression and enhanced apoptosis of fungal cells, highlighting curc-np aPI as a potential treatment for *T*. *rubrum* skin infections.

## Introduction

Dermatophytic fungi utilize nutrients from keratinized tissue, such as skin, hair and nails, and are the etiologic agents of superficial skin mycoses, known as dermatophytoses [1]. The incidence of dermatophytoses has increased due to the growing number of immunocompromised individuals and rising antimicrobial resistance rates [2–4]. Fungal resistance has been particularly pronounced for *Trichophyton rubrum*, the most common organism implicated in cutaneous fungal infections [5, 6], and the cause of invasive and recurrent infections in immunocompromised patients [7]. Currently utilized therapeutics effectively target metabolically active organisms but do not eliminate arthroconidia and dormant spores, leading to treatment failure despite systemic therapy [8].

Given the superficial nature of these infections and ease of access by a light source, there has been renewed focus on antimicrobial photodynamic inhibition (aPI) for fungal infections [9, 10, 11]. aPI is a technique that generates reactive oxygen (ROS) and nitrogen species (RNS) by exciting a pharmacologically inert photosensitizer (PS) with light matched to its absorption wavelength, in the presence of oxygen [12, 13]. In its activated state, the PS can undergo two photochemical reactions: Type I and Type II. The Type I mechanism involves transfer of electrons to a substrate that reacts with oxygen to produce intermediates such as superoxide (O_2_^•−^), hydrogen peroxide and lipid-derived radicals. The Type II reaction is more common and involves direct transfer of electrons to ground-state molecular oxygen to produce excited-state singlet oxygen (^1^O_2_) [11, 14, 15]. In contrast to conventional antibiotics that target a single pathway, ROS and RNS damage multiple cellular structures, limiting the development of resistance [12, 13, 15].

PSs that have been evaluated against dermatophytes include dyes from the porphyrin and phenothiazine classes [10]. These agents have demonstrated the susceptibility of dermatophytes to aPI therapy; however, few studies explore the mechanism of action against *T*. *rubrum*. In addition, other PSs with different properties and structures, such as curcumin, have not been utilized. Curcumin (diferuloylmethane) is a yellow crystalline compound isolated from the spice turmeric that has emerged as a potential photosensitizing compound [16–26]. Curcumin absorbs in the 408–434 nm range [17], and requires blue light for photoactivation. However, its therapeutic translation has been limited by poor aqueous solubility and rapid degradation at physiologic pH, creating a formulation challenge [17].

Encapsulation in nanoparticles has the potential to stabilize curcumin from degradation and allow for suspension in an aqueous solvent [27]. Liposomes, cyclodextrins and micelles have been investigated as solubilizers and nanocarriers of curcumin for aPI against certain bacterial species [18, 20, 28, 29]. However, these methods are hindered by preferential attraction of curcumin to the carrier rather than microbial surfaces and temporal instability, and, therefore, decreased efficacy following preparation. In the present study, a hydrophilic matrix, which swells to release curcumin in an aqueous environment was used to overcome these limitations. We compared photoactivated free curcumin (curc) and encapsulated curcumin (curc-np) against *T*. *rubrum*. The aim of the study was to evaluate curcumin as an effective PS against dermatophytes as well as to determine its mechanism of action.

## Materials and Methods

### Preparation of inoculum

The clinical strain, *T*. *rubrum* BR1A, was obtained with written patient consent according to the institutional review board at Montefiore Medical Center. The inoculum was prepared according to Santos et al [30].

### Synthesis of curcumin-nanoparticles

To create curc-np, we modified our previously described sol-gel-based protocol [31, 32]. Tetramethyl orthosilicate (TMOS) was hydrolyzed by adding HCl, followed by sonication on ice. The mixture was refrigerated at 4°C until monophasic. Curcumin was dissolved in methanol and combined with chitosan (4.4%), polyethylene glycol (4.4%) and TMOS-HCl (8.8%) to induce polymerization. The gel was lyophilized at ∼200 mTorr for 48–72 hours. The resulting powder was processed in a ball mill for ten 30-minute cycles to achieve smaller size and uniform distribution. Complete characterization of curc-np was performed and published previously [33]. Control nanoparticles (control-np) were synthesized identically but without the incorporation of curcumin.

### Preparation of curc and curc-np photosensitizers

A curcumin (Sigma-Aldrich, St. Louis, MO, USA) stock solution was prepared at a concentration of 200 mg/mL in 100% of DMSO. For susceptibility testing, the stock was dilution in RPMI 1640 medium to a final concentration of 40 μg/mL. For aPI, the stock was diluted in PBS to concentrations of 1.0, 10 and 100 μg/mL. The final concentration of dimethyl sulfoxide (DMSO) in both dilutions was less than 1%, such that the solvent did not contribute to observed fungicidal activity. A comparative concentration of curcumin incorporated in nanoparticles was used based on spectrophotometric release curves showing that each mg of curc-np contained 10 μg of curcumin [33]. For susceptibility testing, 8 mg of curc-np was suspended in 1 mL of PBS and diluted in RPMI to a final concentration of 4.0 μg/mL (equivalent to 40 μg/mL of encapsulated curcumin). For aPI, 10 mg of curc-np was suspended in 1 mL of PBS and serially diluted to obtain 100 μg/mL, 1.0 mg/mL and 10 mg/mL of curc-np (equivalent to 1.0, 10, and 100 μg/mL of encapsulated curcumin).

### Light source

The light source used was BLU-U light model 4070 (DUSɅ pharmaceuticals, Wilmington, MA, USA), which emits blue light at a wavelength of 417 ± 5 nm. The doses used were 10 J/cm^2^ (17 minutes), 20 J/cm^2^ (34 minutes), and 40 J/cm^2^ (68 minutes).

### aPI testing

aPI was performed according to Baltazar et al [12]. For aPI optimization, fungal cells were submitted to different treatment conditions by varying the PS concentration and light dose, as described in Table 1. PS without photoactivation and blue light alone served as dark toxicity and light controls, respectively. A 1% DMSO solution in control medium was evaluated for any contributing toxicity.

**Table 1:** Groups and conditions for performing antimicrobial photodynamic therapy.

Groups	Treatments
Controls	
Untreated control (C)	*T*. *rubrum* microconidia only
Blue light (B.L.)	*T*. *rubrum* microconidia irradiated with blue light 417 ± 5 nm.
curcumin 0.1 μg/mL,	*T*. *rubrum* microconidia treated with curcumin 0.1 μg/mL for 10 minutes under light protection.
curcumin 1.0 μg/mL,	*T*. *rubrum* microconidia treated with curcumin 1.0 μg/mL for 10 minutes under light protection.
curcumin 10 μg/mL,	*T*. *rubrum* microconidia treated with curcumin 10 μg/mL for 10 minutes under light protection.
curc-np 0.1 μg/mL,	*T*. *rubrum* microconidia treated with curc-np 0.1 μg/mL for 10 minutes under light protection.
curc-np 1.0 μg/mL,	*T*. *rubrum* microconidia treated with curc-np 1.0 μg/mL for 10 minutes under light protection.
curc-np 10 μg/mL,	*T*. *rubrum* microconidia treated with curc-np 10 μg/mL for 10 minutes under light protection.
Blue light 10 J/cm^2^	*T*. *rubrum* microconidia irradiated with blue light dose of 10 J/cm^2^
Blue light 20 J/cm^2^	*T*. *rubrum* microconidia irradiated with blue light dose of 20 J/cm^2^
Blue light 40 J/cm^2^	*T*. *rubrum* microconidia irradiated with blue light dose of 40 J/cm^2^
Treatments	
curcumin + Blue light 10 J/cm^2^	*T*. *rubrum* microconidia treated with curcumin 10 μg/L, for 10 minutes under light protection, followed by irradiation with blue light dose of 10 J/cm^2^.
curcumin + Blue light 20 J/cm^2^	*T*. *rubrum* microconidia treated with curcumin 10 μg/L, for 10 minutes under light protection, followed by irradiation with blue light dose of 20 J/cm^2^.
curcumin + Blue light 40 J/cm^2^	*T*. *rubrum* microconidia treated with curcumin 10 μg/mL for 10 minutes under light protection, followed by irradiation with blue light dose of 40 J/cm^2^.
curc-np + Blue light 10 J/cm^2^	*T*. *rubrum* microconidia treated with curc-np 10 μg/mL for 10 minutes under light protection, followed by irradiation with blue light dose of 10 J/cm^2^.
curc-np + Blue light 20 J/cm^2^	*T*. *rubrum* microconidia treated with curc-np 10 μg/mL for 10 minutes under light protection, followed by irradiation with blue light dose of 20 J/cm^2^.
curc-np + Blue light 40 J/cm^2^	*T*. *rubrum* microconidia treated with curc-np 10 μg/L, for 10 minutes under light protection, followed by irradiation with blue light dose of 40 J/cm^2^.

### Susceptibility testing and aPI growth curve

Susceptibility of *T*. *rubrum* to ground-state curcumin was tested by a microdilution method according to CLSI M38-A [30, 34]. The itraconazole concentration ranged from 0.015 μg/mL to 8 μg/mL and curcumin and curc-np concentrations from 0.0012 μg/mL to 20 μg/mL. A 1% DMSO solution in control medium was evaluated. The MIC value was defined as the concentration required for 80% fungal growth compared to untreated control [12, 30]. Growth kinetics of ground-state curcumin compared to aPI was also evaluated. Growth was evaluated for 7 days at 28°C using Bioscreen C growth curve system (Growth Curves USA, Piscataway, NJ, USA).

### Measurement of reactive oxygen and nitrogen species

Intracellular generation of ROS and RNS was evaluated using 50 μM of 2′,7′dichlorodihydrofluorescein diacetate (H_2_DCFDA, Invitrogen) to quantify ROS, 10 μM of 4-amino-5-methylamino-2′,7′-difluorofluorescein (DAF-FM, Invitrogen) to quantify NO^•^, and 50 μM dihydrorhodamine 123 (DHR 123, Invitrogen) to quantify ONOO^−^. Following aPI, samples were incubated with fluorescent probes for 30 minutes at 28°C [12], and subsequently analyzed with flow cytometry (Becton Dickinson LSRII, USA) using a 530/30 nm band pass filter for fluorescence detection. The Mean Fluorescence Intensity (MFI) was considered to determine radical production. Data analyses were performed using FlowJo 10.1 software.

### Treatment with ROS and RNS scavengers

Different ROS and RNS scavengers were used to evaluate the effect of radical stress inhibition on aPI efficacy. The scavengers included: 5,10,15,20-tetrakis-(4-sulfonatophenyl)-porphyrinato iron (III) chloride (FeTPPs) (1 and 0.1 mM, Calbiochem) as a ONOO^−^ scavenger, 4,5-dihydroxy-1,3-benzenedisulfonic acid disodiumsalt hydrate (Tiron) (1.0 and 10 mM, Sigma-Aldrich, St. Louis, MO, USA) as a O_2_^•−^ scavenger, sodium pyruvate (0.1, 1.0 and 10 mM, Sigma-Aldrich) as a hydrogen peroxide scavenger, carboxy-PTIO (0.2 and 2.0 mM, Cayman chemical, Ann Arbor, MI, USA) as a NO^•^ scavenger, D-mannitol (100 mM, Sigma-Aldrich) as a hydroxyl radical scavenger and sodium azide (1.0, 10 mM and 1.0 M, Sigma-Aldrich) as an ^1^O_2_ scavenger. Scavengers were added to fungal suspensions immediately before initiation of aPI and incubated for 1 h with RPMI 1640 without phenol red plus 2% glucose at 28°C. To evaluate fungal viability, 150 μL of the fungal suspensions were plated onto PDA, and incubated at 28°C for 72 hours [12].

### Apoptosis assay

The HT TitierTACS assay kit (Trevigen, Gaithersburg, MD, USA) was used to evaluate the occurrence of apoptosis after aPI. The assay was performed according to manufacturer’s instructions.

### Phagocytosis assay

J774.16 macrophages were grown at 37°C with 10% CO_2_ in DMEM (Cellgro, Manassas, VA, USA), supplemented according to Guimarães et al [35]. The fungal-macrophage cell proportion was 1:1 [36], with 5.0x10^5^ fungal cells to 5.0x10^5^ macrophage cells. After challenging macrophages with *T*. *rubrum* microconidia, the cells were submitted to aPI, followed by incubation in the 10% CO_2_ chamber at 37°C for 24 hours. The macrophages were lysed with cold water and the lysate plated onto PDA and incubated at 28°C for 72 hours.

### Ethics approval

#### Human ethics

The clinical strain, *T*. *rubrum* BR1A, was obtained with written patient consent and the use approved by the institutional review board at Montefiore Medical Center.

### Statistical analysis

Statistical analyses were performed with GraphPad Prism software using one-way analysis of variance (ANOVA), Newman-Keuls multiple comparison tests or Student’s *t-*test, according to the data.

## Results

### Antimicrobial photodynamic inhibition

**Fig 1:**
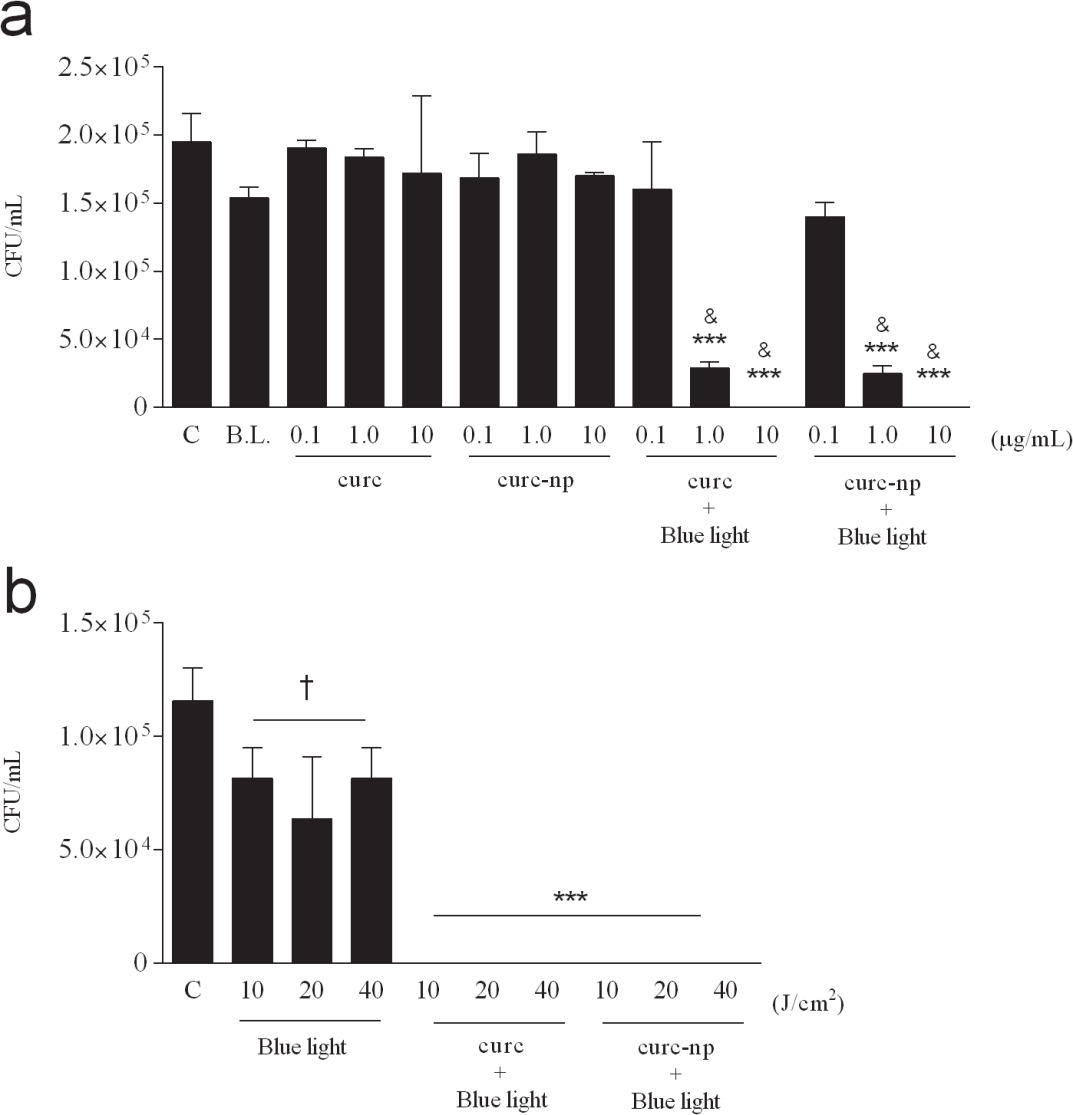
Optimization of aPI conditions. **(a)** Effect of varying the PS concentration on fungal growth, as determined by colony forming units (CFU), using a constant light source of 40 J/cm^2^. **(b)** Effect of varying the light dose using a constant PS concentration of 10 μg/mL. Untreated *T*. *rubrum* (C), Blue light alone (B.L.) and PS without photoactivation (curc and curc-np) were used as controls. ***Compared to untreated, blue light and PS without photoactivation. ^&^Compared to lowest PS concentration of same group. ^†^Compared to untreated control. ***p < 0.0001, ^&, †^p < 0.05. Data are a composite of three independent experiments with each treatment group performed in triplicate. The results are expressed as the mean ± SEM.

A range of curcumin concentrations and blue light doses were evaluated to determine the efficacy of curcumin as a PS and to define the optimal treatment conditions (Table 1). At a light dose of 40 J/cm^2^, concentrations of 1.0 and 10 μg/mL of curc and curc-np significantly decreased fungal viability in a dose dependent manner compared to untreated control (p<0.0001), with the highest concentration achieving complete growth inhibition (Fig. 1A). The lowest PS concentration (0.1 μg/mL) did not differ significantly from untreated control. PS without photoactivation did not reduce fungal burden at the three concentrations tested (p<0.05), nor did the 1% DMSO solution (data not depicted). In combination with the most effective PS concentration (10 μg/mL), all three blue light doses completely inhibited *T*. *rubrum* growth (p<0.0001, Fig. 1B) and were significant compared to untreated and blue light controls. Blue light alone, without the addition of PS, exhibited fungicidal activity (p<0.05), but did not completely inhibit growth, with no differences observed between light fluences. Based on these results, a PS concentration of 10 μg/mL combined with a blue light dose of 10 J/cm^2^ were chosen for all subsequent analyses.

### Susceptibility testing and growth kinetics

The intrinsic antifungal activity of ground-state curcumin was evaluated by incubating *T*. *rubrum* with a range of curc and curc-np concentrations without photoactivation (data not represented). Seven-day incubation with ground-state curcumin did not yield significant 80% reduction of fungal growth. A 1% DMSO solution did not exert any fungicidal activity (data not represented). Itraconazole was used as a comparative control to test the virulence of the clinical *T*. *rubrum* strain. The MIC value of itraconazole was 0.25 μg/mL, which is within the reported range [37], increasing the generalizability of our findings.

**Fig 2:**
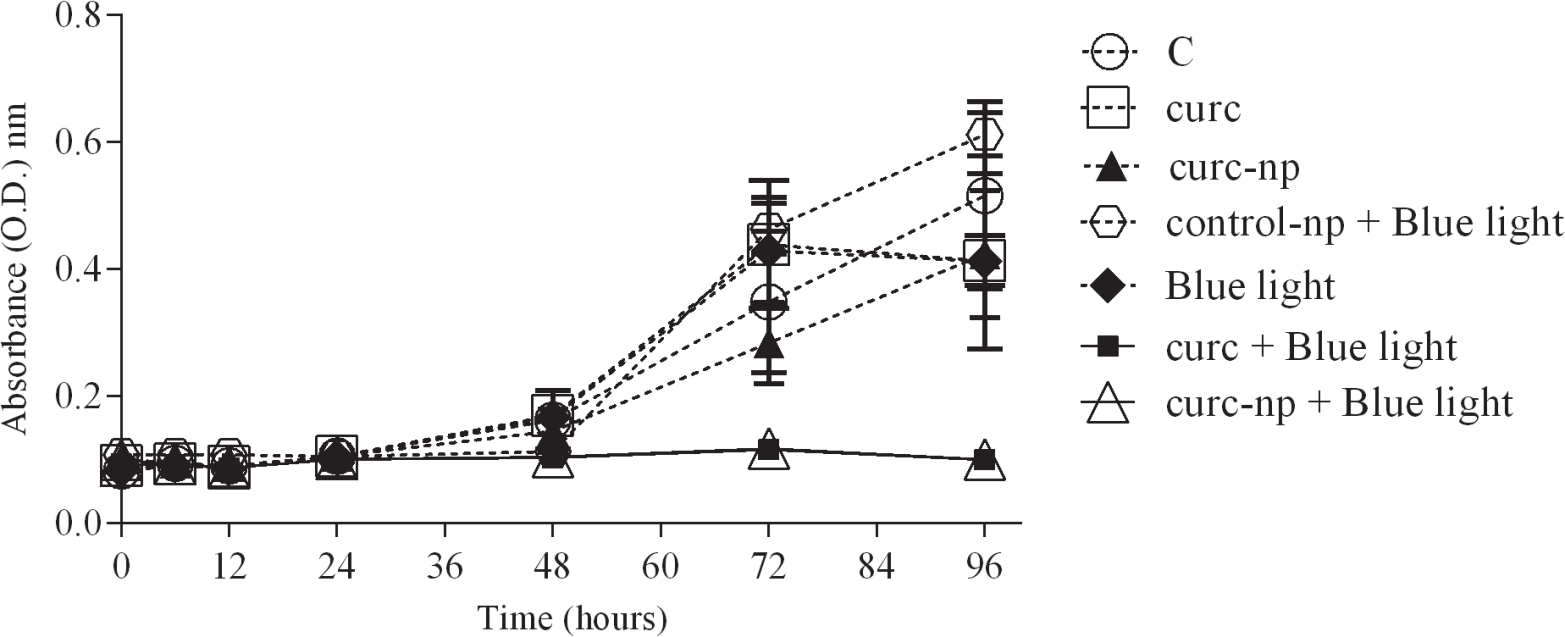
Fungal growth curve after aPI. Fungal growth curve of aPI at optimal conditions (10 μg/mL of PS with 10 J/cm^2^ of B.L.). Each treatment per group was performed in triplicate and data are a composite of three independent experiments. The results are expressed as the mean ± SEM.

Differences in growth kinetics between *T*. *rubrum* treated with ground-state and photoactivated curcumin were observed at 48 hours of incubation (Fig. 2). A steady increase of growth was observed for the PS control, while aPI completely inhibited growth for the full seven days (represented until 96 hours). Control-np (10 μg/mL) alone and in combination with 10 J/cm^2^ of blue light did not impact fungal growth compared to untreated control.

### Measurement of ROS and RNS

**Fig 3:**
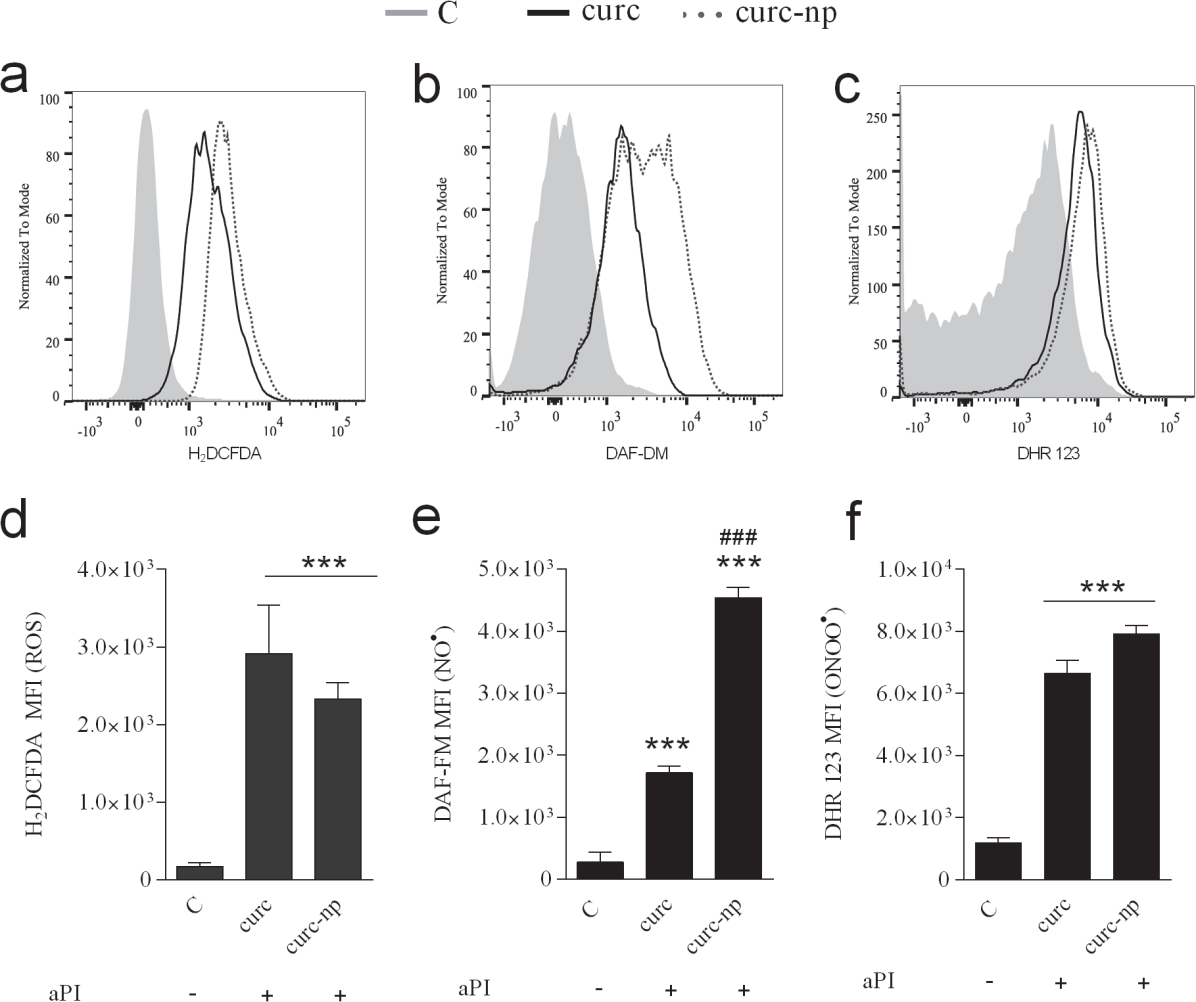
Evaluation of ROS and RNS production after aPI. Detection of ROS levels following aPI, expressed as a **(a)** representative histogram and **(d)** cumulative bar plot. Detection of NO^•^ levels following aPI, expressed as a **(b)** representative histogram and **(e)** cumulative bar plot. Detection of ONOO^−^ levels following aPI, expressed as a **(c)** representative histogram and **(f)** cumulative bar plot. Dark toxicity controls did not differ significantly from untreated *T*. *rubrum* (data not represented). ***Compared to untreated control. ^###^Compared to curc group. **MFI**. Mean fluorescence intensity. ***,^###^p < 0.0001. Each treatment per group was performed in triplicate and are a composite of two independent experiments. The results are expressed as the mean ± SEM.

The levels of ROS and RNS after aPI were evaluated using probes to quantify reactive species generation. Compared to untreated control, photoactivated curcumin induced a significant increase in the generation of both reactive oxygen and nitrogen radicals (p<0.0001, Fig. 3A-F). Treatment with curc and curc-np induced a fold-change in ROS production by 17 and 13, respectively (Fig. 3A and D). For NO^•^ production, a greater disparity between curc and curc-np was observed, with a fold change of 6 and 16, respectively (Fig. 3B and E). Measurement of ONOO^−^ production following treatment with curc and curc-np demonstrated the smallest fold-change of 7 and 6, respectively (Fig. 3C and F).

### Treatment with ROS and RNS scavengers

**Fig 4:**
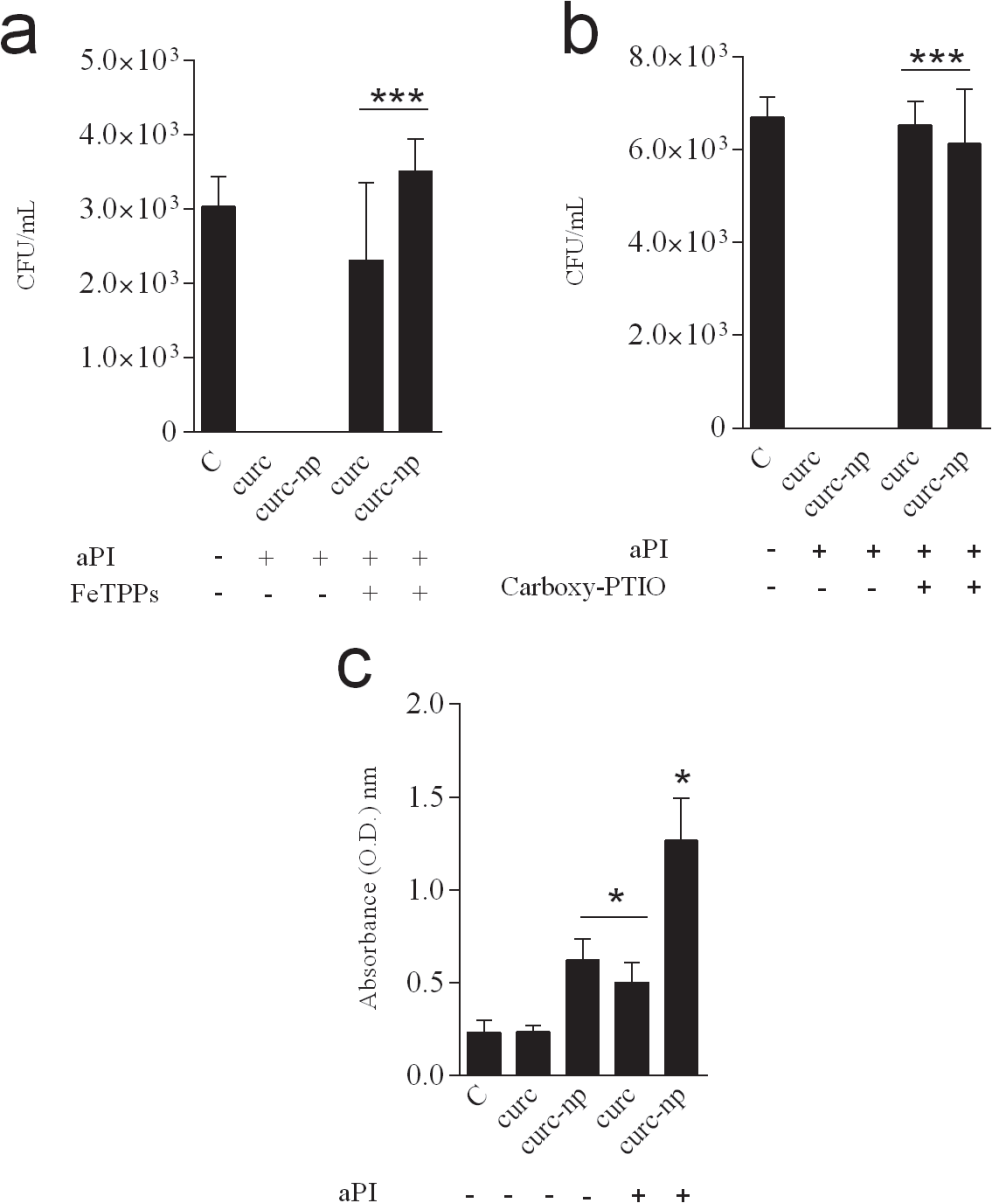
Evaluation of aPI mechanism of action. **(a)** Treatment with ONOO^−^ scavenger (FeTPPs). **(b)** Treatment with NO^•^ scavenger (Carboxy-PTIO). **(c)** Apoptosis assay performed after aPI. ***Compared to aPI treatment in the absence of incubation with scavengers. *Compared to untreated *T*. *rubrum* control. *p< 0.05, ***p< 0.0001. Each treatment per group was performed in triplicate and data are a composite of two independent experiments. The results are expressed as mean ± SEM.

Scavengers of ROS and RNS were used to evaluate the role of different radicals in aPI. None of the concentrations of Tiron (superoxide anion scavenger), sodium pyruvate (hydrogen peroxide scavenger), D-mannitol (hydroxyl radical scavenger) or sodium azide (singlet oxygen) inhibited aPI efficacy. Despite pre- incubation with these compounds, no fungal growth was observed in any of these groups (data not shown). Interestingly, only incubation with RNS scavengers, particularly FeTPPs (ONOO^−^ scavenger) and carboxy-PTIO (NO^•^ scavenger) interfered with aPI activity (Fig. 4A and B). *T*. *rubrum* growth was relatively intact despite aPI in the presence of the highest concentrations tested of FeTPPs (1.0 mM) and carboxy-PTIO (2.0 mM). The apoptosis assay showed that curc alone did not induce apoptosis of *T*. *rubrum* cells compared to untreated control; however, after irradiation with blue light, there was a significant trend towards increased apoptosis (p<0.05, Fig. 4C). Curc-np, on the other hand, significantly increased the occurrence of apoptosis in comparison to untreated control (p<0.05). Additionally, an extreme augmentation of apoptotic fungal cells was observed after treatment with curc-np in combination with blue light (p<0.0001).

### Phagocytosis assay

**Fig 5:**
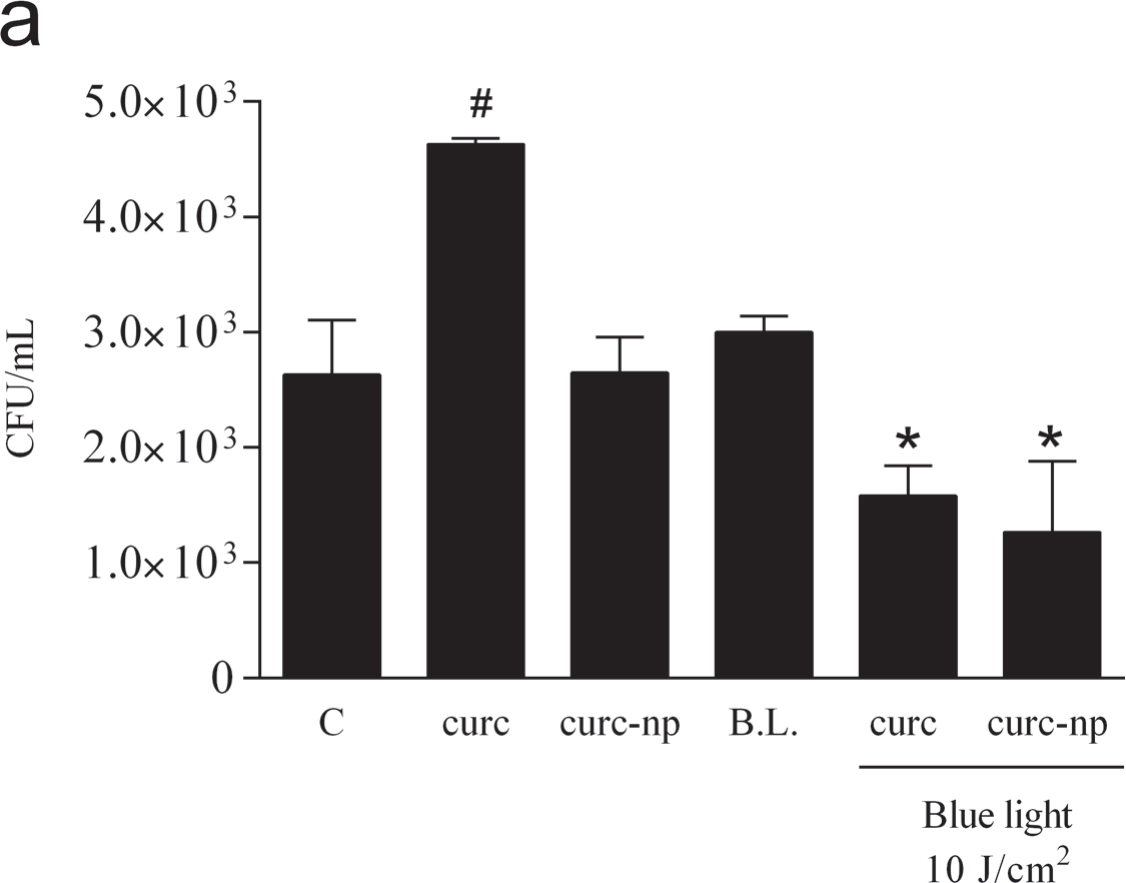
Phagocytosis assay. CFU quantification of macrophages challenged with *T*. *rubrum* cells and treated with aPI therapy. ^#^ Compared to untreated control (C), dark toxicity and blue light 10 J/cm^2^ (B.L.) controls. * Compared to all other groups. B.L. Blue light 10 J/cm^2^ (17 minutes). *,^#^ p < 0.05. Each treatment per group was performed in triplicate and data is a composite of two independent experiments. The results are expressed as the mean ± SEM.

Macrophages were challenged with *T*. *rubrum* and treated with aPI to investigate the efficacy against infected mammalian cells. aPI with curc and curc-np significantly reduced fungal burden compared to untreated, dark toxicity and blue light controls (p<0.05, Fig. 5). Ground-state curcumin had a protective effect and caused a decrease in macrophage-induced destruction of *T*. *rubrum* cells (p<0.05).

## Discussion

This is the first study to evaluate the susceptibility of *T*. *rubrum* to the photosensitizer curcumin in conjunction with blue light and analyze its mechanism of action. Ground-state curcumin or blue light alone did not inhibit fungal growth, affirming the combination of light and PS necessary for aPI activity. Though other studies attribute innate antifungal properties to ground-state curcumin [38–40], in the present work none of the concentrations evaluated were fungicidal. This discrepancy may result from differences in curcumin concentrations, as well as purity. Wuthi-udomlert et al [40] showed that oil isolated from *Curcuma longa* exhibited antifungal effects against dermatophytes at MIC values of 7.8 and 7.2 mg/mL respectively. With photoactivation, we elicited toxic effects using only 10 μg/mL of curcumin, a low dose with no intrinsic microbicidal activity as seen with MIC susceptibility testing.

BLU-U light was chosen as the light source due to its resonance with curcumin. It is used clinically to treat patients with *Propionibacterium acnes* infection at a light fluence of 10 J/cm^2^, which is equivalent to the dose used in this investigation [41]. Blue light therapy has not been associated with histologic signs of epidermal or dermal DNA damage, photoaging and inflammation *in vivo* [42]. Some *in vitro* investigations have demonstrated dose-dependent toxicity to mammalian cells but at higher light fluences than used in the present study [43–45].

Improvement of aPI performance through the use of nanotechnology has been investigated for curcumin, though not against *T*. *rubrum*. Compared to free form, nanoencapsulation protects curcumin from hydrolytic and enzymatic degradation and enhances its delivery due to increased aqueous solubility [20]. In addition, nanoparticles reduce PS aggregation, which is known to decrease photodynamic reactivity, and can promote selectivity by passive or active targeting. In this study, encapsulation in a hydrophilic matrix was used to overcome the limitations observed with other formulations [18, 20, 21, 46] and allows for sustained release, important for topical drug delivery.

The phototoxic effects of activated curcumin in microbial systems is thought to be oxygen dependent, involving the reaction of excited states of curcumin with oxygen to generate ROS and RNS [17,19]. The direct contribution of a Type II ^1^O_2_ reaction to the observed toxicity of photoactivated curcumin is controversial. Shen et al [47] showed curcumin photosensitization to yield both ^1^O_2_ and O_2_^•−^ radicals; however, other studies have found no evidence of ^1^O_2_ formation [20, 23]. In accordance with our findings, aPI executed in the presence of sodium azide was not associated with enhanced fungal growth [26], suggesting the effects of curcumin aPI are not mediated by ^1^O_2_ directly, but via the generation of downstream radicals, such as ROS and RNS. In the present study, among several scavengers of ROS and RNS tested, only FeTPPS and Carboxy-PTIO blocked aPI efficacy and resulted in *T*. *rubrum* growth, pointing to a Type I, primarily nitrosative pathway. Production of nitric oxide involves the catabolism of L-arginine by nitric oxide synthases (NOSs) into citrulline and NO^•^ [12]. However, further work is required to elucidate the exact biochemical source of NO^•^ (enzymatic or non-enyzmatic) after aPI. Reactions of NO^•^ with O_2_^•−^ produces ONOO^−^, which can then form additional RNS, including nitrogen dioxoide and dinitrogen trioxide. Nitric oxide-mediated antimicrobial action occurs by a variety of mechanisms, including DNA damage, peroxidation of lipid membranes, and S-nitrosylation of thiol residues [48–50]. Peroxynitrite has greater cytotoxic potential than NO^•^ or O_2_^•−^ alone and further accelerates cellular degradation [48, 51].

In association with the augmentation of ROS and RNS production, the present work shows that curcumin aPI induces apoptosis of *T*. *rubrum* cells. The reactive radicals produced by aPI initiate a cascade of biochemical events, which damage multiple cellular compartments, culminating in cell death via apoptosis [11–13, 52]. Sharma et al [53] similarly reported that photoactivated curcumin raised ROS levels, triggering early apoptosis in *Candida albicans*. The curc-np group was associated with significantly more apoptosis than free curcumin, suggesting that, at conditions evaluated, encapsulated curcumin induces stronger DNA fragmentation than free curcumin. It is possible that the higher surface to volume ratio of nanoparticles allows for enhanced interaction and improved PS availability, thus initiating a more intense apoptotic effect. As observed, the curc-np induced a remarkable augmentation of NO^•^ levels, which can lead to organelle dysfunction and signalization of apoptosis [51]. The experimental model using macrophages emphasizes the clinical translatability of our work. Macrophages act through the induction of oxidative and nitrosative stress and the enhanced generation of radicals by curcumin aPI can amplify host macrophage activity [14]. Ground-state curc had a protective effect on *T*. *rubrum* cells; possibly due to the antioxidative nature of curcumin, which can mitigate a macrophage-induced ROS environment.

In conclusion, the present study shows curcumin aPI to be an effective alternative for the treatment of *T*. *rubrum* infection. The curc-np outperformed free curcumin by generating greater NO^•^ levels with subsequently increased apoptosis and the ease of formulation and use makes clinical translation of the curc-np formulation promising. Photodynamic therapy is ideal for topical applications, wherein the combined treatment of light and PS can be targeted to a distinct lesion, minimizing adjacent toxicity. The reactive radicals generated by curcumin aPI are not prone to the development of resistance and act close to the site of their generation, supporting the use of aPI in clinical practice. Our laboratory is currently investigating and optimizing an *in vivo* infection model of *T*. *rubrum*. Recalcitrant and deep infections, often in immunocompromised hosts, require treatment with weeks of systemic antifungals [54] and the potential to treat topically is a huge advance from the current approach. Preliminary *in vivo* studies suggest that curc-np has a predilection for the hair follicle (S1 Fig.)—the clinical site of *T*. *rubrum* infection, such as Majocchi’s granuloma—which can further allow for enhanced targeting and decreased contiguous toxicity. The data provided in this manuscript highlights aPI as a promising new avenue for the treatment of dermatophyte infections and an area of significant interest for topical use.

## Supporting Information

S1 Fig Curc-np penetration of hair follicles. Histologic imaging of tissue after application of fluorescent curc-np for 30 minutes without occlusion showed preferential accumulation within hair follicles. Fluorescent curc-np were synthesized by conjugation to Alexa-fluor 494 dye during synthesis. 5mm punch biopsies were taken and tissue embedded in OCT. 10-m thick sections were cut using a cryostat and mounted onto glass slides. All skin sections were stained with hematoxylin and eosin (H&E). Photographs of the skin sections were taken using light and fluorescence microscopy and the images merged to localize the fluorescent nanoparticles.
(TIF)
