# Supplementary figures and images for: Trichophyton rubrum is Inhibited by Free and Nanoparticle Encapsulated Curcumin by Induction of Nitrosative Stress after Photodynamic Activation
Source: PLoS One. 2015 Mar 24;10(3):e0120179. doi: 10.1371/journal.pone.0120179 (PMC4372525; doi:10.1371/journal.pone.0120179)

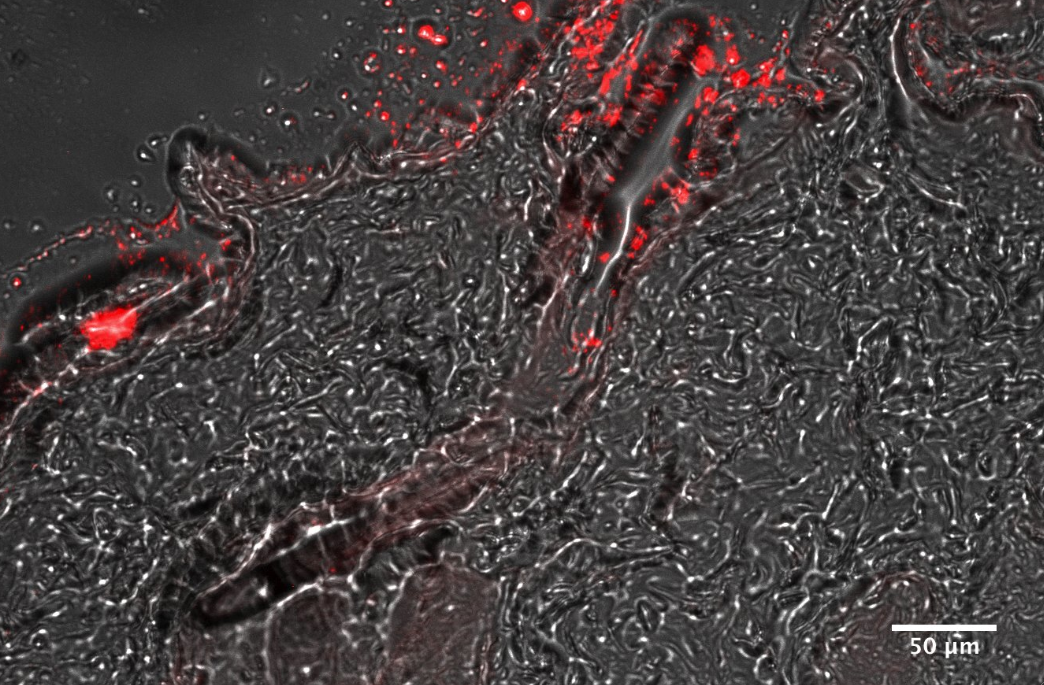

Supplement: S1 Fig — Histologic imaging of tissue after application of fluorescent curc-np for 30 minutes without occlusion showed preferential accumulation within hair follicles. Fluorescent curc-np were synthesized by conjugation to Alexa-fluor 494 dye during synthesis. 5mm punch biopsies were taken and tissue embedded in OCT. 10-m thick sections were cut using a cryostat and mounted onto glass slides. All skin sections were stained with hematoxylin and eosin (H&E). Photographs of the skin sections were taken using light and fluorescence microscopy and the images merged to localize the fluorescent nanoparticles. (TIF) [file pone.0120179.s001.tif]
